# Fatal Intracerebral Hemorrhage Following Carotid Artery Stenting: A Case Report

**DOI:** 10.1002/ccr3.72026

**Published:** 2026-02-10

**Authors:** Linling Ji, Jiahao Wang, Yanyan Ji, Lin Zhu, Yingge Wang

**Affiliations:** ^1^ The First School of Clinical Medicine Faculty of Medicine Yangzhou University Yangzhou China

**Keywords:** carotid artery stenting, carotid Hyperperfusion syndrome, case report, intracranial hemorrhagepostoperative management and intervention

## Abstract

This case highlights the importance of individualized treatment decisions in patients with recently symptomatic carotid artery stenosis causing cerebral infarction, including recurrent events within 1 year and a new infarction occurring 18 days before intervention. It also emphasizes the need for early recognition and timely management of postoperative carotid hyper perfusion syndrome.

## 1 Introduction

Carotid hyper perfusion syndrome (CHS) is a rare but devastating complication of carotid revascularization, occurring after carotid endarterectomy (CEA) or carotid artery stenting (CAS). The pathogenesis of CHS is primarily related to impaired cerebrovascular autoregulation, hemodynamic changes in cerebral blood flow following revascularization, oxidative stress, and disruption of the blood–brain barrier [1, 2]. CHS can cause a variety of symptoms, including headache, seizures, focal motor weakness, and intracranial hemorrhage. The mortality rate associated with CHS‐induced intracranial hemorrhage is high, and survivors often experience significant neurological deficits [3, 4]. Therefore, early identification of high‐risk patients for postoperative CHS, timely intervention, and meticulous postoperative management are critical to improving outcomes [5]. In this report, we describe a case of severe CHS following intracranial stenting for carotid artery stenosis, characterized by a rapid increase in blood pressure and progression of symptoms shortly after surgery.

## 2 Case Presentation

### 2.1 Case History and Examination

A 61‐year‐old male patient with a medical history of hypertension, type 2 diabetes mellitus, cerebral infarction, cerebral hemorrhage, and liver cirrhosis was admitted due to symptomatic severe internal carotid artery stenosis. Six months prior, he was diagnosed with acute cerebral infarction following an episode of numbness and weakness in his right limbs, which improved with treatment. After discharge, the patient continued long‐term antiplatelet therapy and statin medications to stabilize plaques and prevent further cerebral infarctions.

**FIGURE 1:**
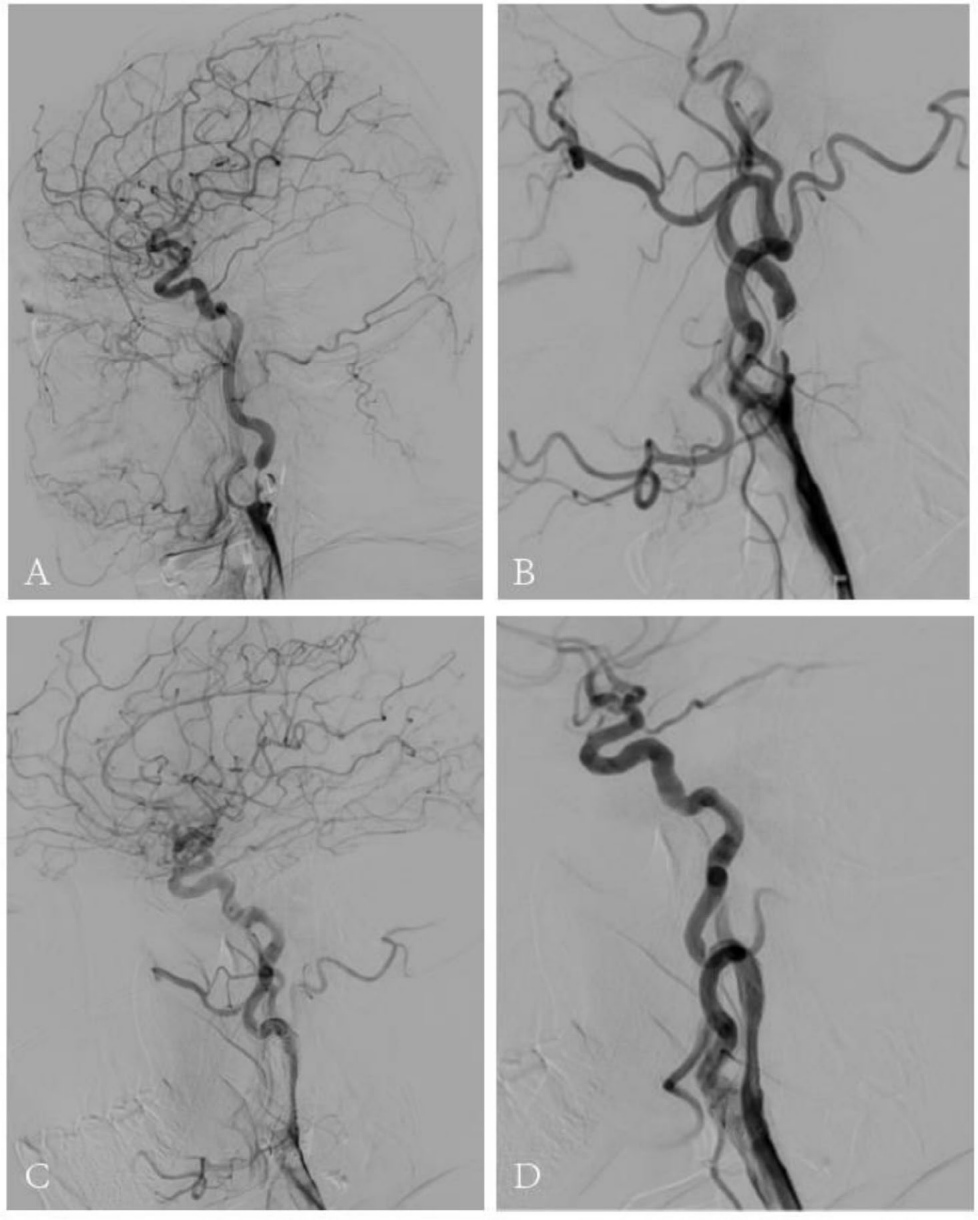
Cerebral angiography images of the patient with internal carotid artery stenting: (A–B): Preoperative angiography shows severe stenosis at the origin of the right internal carotid artery (C1 segment) and at the origin of the left internal carotid artery (C1 segment). (C–D): Intraoperative angiography demonstrates successful stent placement with a residual stenosis of 15%.

One week before admission, he developed slurred speech and right limb weakness without an obvious precipitating factor. Neurological examination revealed grade 4 muscle strength in the right limbs and positive pathological signs on the right side. Imaging confirmed a new acute cerebral infarction, while cerebral angiography demonstrated severe stenosis at the origin (C1 segment) of both the right and left internal carotid arteries (Figure 1 A–B). The patient had two episodes of acute ischemic stroke within the previous 6 months. Cerebral angiography showed severe stenosis at the origins of both internal carotid arteries with occlusive changes, suggesting a high risk of recurrent ischemic events. After multidisciplinary assessment by the neurology and interventional teams, revascularization was considered necessary.

New ischemic stroke symptoms occurred on March 20 2025. According to the ESVS guidelines, CEA is considered the safer option when intervention is performed within the first 14 days after symptom onset [6]. Beyond this period, outcomes of CEA and CAS are generally comparable [7]. During the patient's second hospitalization, we discussed in detail the feasibility, perioperative risks, and potential benefits of both CAS and CEA. After shared decision‐making with the patient and family, CAS was ultimately selected. CAS was then performed as planned on April 9 2025.

### 2.2 Investigation and Treatment

After admission, the patient underwent comprehensive laboratory and imaging evaluations, which revealed no significant abnormalities and no contraindications to surgery (Table 1). On the morning of the procedure, blood pressure was measured at 148/94 mmHg. Given the possibility that carotid artery stenting (CAS) may stimulate the carotid sinus, potentially leading to bradycardia and hypotension, antihypertensive medications were withheld on the day of the operation.

**TABLE 1:** Blood tests on initial presentation.

Parameter	Result	Reference range	Abnormality
Neutrophil percentage	74.7%	50%–70%	↑
Lymphocyte percentage	17.4%	20%–40%	↓
Platelet count	115 × 10^9^/L	125–350 × 10^9^/L	↓
Hemoglobin	130 g/L	130–175 g/L	Normal
Hematocrit	42.3%	40%–50%	Normal
High‐sensitivity C‐reactive protein (hs‐CRP)	3.29 mg/L	0–6.0 mg/L	Normal
White blood cell count	7.23 × 10^9^/L	3.5–9.5 × 10^9^/L	Normal
Total bilirubin	24.5 μmol/L	3–22 μmol/L	↑
Indirect (unconjugated) bilirubin	18.8 μmol/L	0–19 μmol/L	↑
AST (aspartate aminotransferase)	27 U/L	0–50 U/L	Normal
ALT (alanine aminotransferase)	21 U/L	17–59 U/L	Normal
γ‐GT (GGT, gamma‐glutamyl transferase)	36 U/L	15–73 U/L	Normal
Lactate dehydrogenase (LDH)	197 U/L	101–240 U/L	Normal
Creatine kinase (CK)	82 U/L	38–126 U/L	Normal
CK‐MB	3.2 U/L	0–16 U/L	Normal
Troponin I (hs‐cTnI)	2.9 pg/mL	< 17.5 pg/mL	Normal
Uric acid	240 μmol/L	208–428 μmol/L	Normal
Creatinine	50.7 μmol/L	58–110 μmol/L	↓
Blood urea nitrogen (BUN)	3.71 mmol/L	3.2–7.1 mmol/L	Normal
Glucose	7.24 mmol/L	4.1–5.9 mmol/L	↑
Sodium (Na^+^)	144.9 mmol/L	137–145 mmol/L	Normal
Potassium (K^+^)	3.08 mmol/L	3.5–5.1 mmol/L	Low
Chloride (Cl^−^)	105.4 mmol/L	98–107 mmol/L	Normal
B‐type natriuretic peptide(BNP)	55 pg/mL	0–100 pg/mL	Normal
Total cholesterol	2.05 mmol/L	2.33–5.69 mmol/L	Low
Triglycerides	0.84 mmol/L	0.23–1.70 mmol/L	Normal
High‐density lipoprotein (HDL)	0.68 mmol/L	1.16–1.55 mmol/L	Low
Low‐density lipoprotein (LDL)	1.28 mmol/L	2.7–3.1 mmol/L	Low
Apolipoprotein A (Apo A)	0.91 g/L	1.2–1.6 g/L	Low
Apolipoprotein B (Apo B)	0.47 g/L	0.63–1.25 g/L	Low
Fatty acid (a)	169.39 mg/L	0–300 mg/L	Normal
Small dense LDL cholesterol	0.62 mmol/L	0.246–1.362 mmol/L	Normal
HCY	12.73 umol/L	5.08–15.39umol/L	Normal

Under local anesthesia, the patient underwent carotid artery stent implantation with cerebral protection. Intraoperatively, severe stenosis of the left internal carotid artery was confirmed. The procedure was uneventful, and significant improvement in the degree of stenosis was achieved postoperatively (Figure 1 C–D). The patient remained hemodynamically stable and returned to the ward after a postoperative cranial CT scan.

Upon returning to the ward, the patient's blood pressure was elevated to 181/78 mmHg. Oxygen therapy, ECG monitoring, and continuous intravenous infusion of urapidil were administered to control blood pressure. Dual antiplatelet therapy and lipid‐lowering treatment were continued.

Approximately 1 h after the procedure, the patient experienced a sudden increase in blood pressure to approximately 210/110 mmHg, followed by severe vomiting of gastric contents. Neurological examination revealed impaired consciousness, bilaterally equal and round pupils with a diameter of 2.0 mm, and sluggish pupillary light reflexes. The remainder of the neurological assessment could not be completed due to lack of patient cooperation. Antiemetic therapy was provided, the urapidil dose was increased, and blood pressure was maintained within the range of 124/77 to 144/89 mmHg.

**FIGURE 2:**
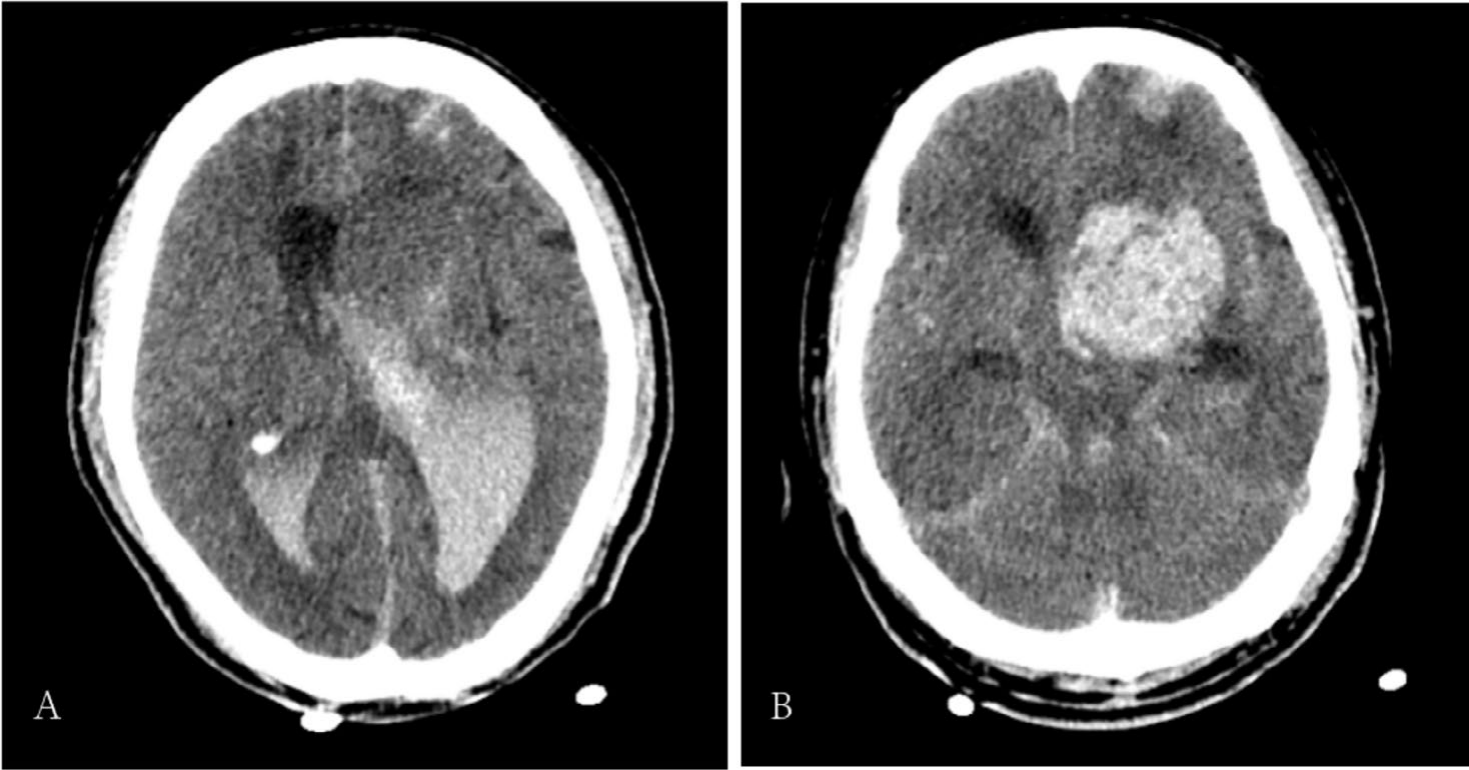
Postoperative cranial CT scan images: (A–B): Two hours after surgery, the cranial CT scan shows a mass‐like high‐density shadow in the left frontal lobe and basal ganglia region. The border is relatively clear, with a size of approximately 5.3 × 4.9 × 2.1 cm. Part of the mass has broken into the ventricles, and the ventricular system is enlarged, with a high‐density cast image observed inside. The midline structure is shifted approximately 0.7 cm to the right.

An emergency cranial CT scan revealed cerebral hemorrhage (Figure 2). Neurosurgical consultation confirmed intracerebral hemorrhage following CAS, consistent with a diagnosis of postoperative hyper perfusion syndrome. The condition was critical, with a high risk of hemorrhage progression, worsening cerebral edema, and possible brain herniation, with an extremely poor prognosis. After discussion with the patient's family, bilateral ventricular drainage or hematoma evacuation was recommended. Following family deliberation, bilateral ventricular drainage was selected and performed successfully.

Postoperatively, the patient remained unconscious and unresponsive to verbal stimuli. No limb movements or seizures were observed in response to orbital pressure‐induced pain. The patient remained intubated and was supported by mechanical ventilation. Both ventricular drainage catheters were unobstructed; however, intracranial pressure was markedly elevated (> 300 mmHg), and there was bloody exudate on the drainage dressings.

Neurological examination revealed shallow coma, bilaterally equal and round pupils measuring 2.5 mm, with absence of pupillary light reflexes. A joint assessment by the neurology and neurosurgery teams suggested a high likelihood of cerebral herniation. Measures to reduce intracranial pressure were intensified, including mannitol therapy, head‐of‐bed elevation, strict blood pressure control, fluid management, sedation and analgesia, and close monitoring of vital signs. The patient's family was informed of the severity of the condition, and further CT imaging and possible decompressive craniectomy were recommended. However, as the patient lacked spontaneous respiration and required ventilator support for transfer, the family declined additional imaging and surgical intervention due to perceived transport risks.

The medical team explained the imminent risk of cardiac and respiratory arrest due to brainstem compression from herniation, and the family acknowledged and understood the prognosis. Despite continued management with osmotic therapy with mannitol, blood pressure control, anti‐infective measures, and nutritional support, the patient's condition continued to deteriorate, and clinical death occurred on postoperative day four.

### 2.3 Outcome

On postoperative day four, the patient's heart rate progressively declined. Blood pressure and oxygen saturation became unmeasurable. The family explicitly declined all resuscitative measures. Bedside electrocardiogram confirmed clinical death.

## 3 Discussion

Cerebral hyper perfusion syndrome (CHS) is a potentially severe complication that may occur following carotid artery stenting (CAS), most commonly within the first 24 h postoperatively [8]. Delayed onset has also been reported, with cases developing up to 1 month after intervention, during which continued monitoring is warranted [9]. Its clinical manifestations include elevated blood pressure, headache, seizures, focal neurological deficits, and intracranial or subarachnoid hemorrhage [10, 11, 12]. Among these, cerebral hemorrhage represents one of the most critical complications, often associated with extremely poor prognosis and a mortality rate exceeding 50% [3, 13]. Although the incidence of such hemorrhage is relatively low and its onset often insidious, failure to recognize and manage CHS in a timely manner can result in serious consequences, including death [3].

The underlying mechanisms of CHS remain unclear, likely involving multiple pathophysiological processes. Among them, impaired cerebrovascular autoregulation and changes in cerebral hemodynamics after revascularization are considered key mechanisms contributing to the development of the syndrome [14]. In patients with long‐term cerebral hypoperfusion (such as those with carotid artery stenosis), chronic compensatory vasodilation maintains cerebral blood flow but leads to a decline in cerebrovascular autoregulatory capacity. When blood flow is suddenly restored (e.g., after carotid endarterectomy or carotid artery stenting), the blood vessels are unable to effectively constrict, resulting in regional hyper perfusion [15]. Following carotid revascularization, the flow velocity in the ipsilateral middle cerebral artery dramatically increases, exceeding the metabolic demands of the brain tissue, which can also lead to hyper perfusion syndrome. Furthermore, oxidative stress, inflammation, and disruptions in pressure‐sensing reflexes may also contribute to the development of CHS. In a hyper perfusion state, if not proactively managed, this can lead to further dysfunction of cerebrovascular autoregulation, endothelial injury, and hemodynamic stress, ultimately resulting in blood–brain barrier disruption, plasma extravasation, and vasogenic edema. In severe cases, this may trigger hemorrhage or even death [16]. Therefore, early prediction and prevention of postoperative hyper perfusion syndrome are crucial for improving patient prognosis.

The risk factors for CHS typically include preoperative long‐term hypertension, diabetes, advanced age, recent stroke history, severe ipsilateral or contralateral carotid stenosis, poor collateral circulation, postoperative hypertension, and the use of antiplatelet or anticoagulant therapies [15]. The progression of HPS to ICH is associated with an extremely poor prognosis, making perioperative blood pressure control critically important [1, 17]. Bouri et al. reported an inflection point in the cumulative incidence of CHS when postoperative systolic blood pressure exceeded 150 mmHg, with 81% of CHS cases occurring at levels above 180 mmHg [18]. In such patients, cerebral autoregulation is impaired and collateral circulation may be limited, making them more vulnerable to ischemia when blood pressure is lowered abruptly. Strict postoperative blood pressure control is therefore important in reducing the risk of hyper perfusion after CAS [19, 20]. Strict postoperative blood pressure control is therefore important in reducing the risk of hyper perfusion after CAS. In the case presented here, the lack of active postoperative blood pressure control led to rapid disease progression.

In addition to strict postoperative blood pressure management, the timing of surgery is also critical. For patients with extensive or progressive ischemic stroke, performing surgery too early may increase the risk of intracranial hemorrhage due to high perfusion [15]. Moreover, patients who have recently undergone contralateral carotid artery surgery have a higher risk of developing CHS. In patients with severe bilateral carotid artery stenosis, the reduced cerebral vascular reserve makes them more prone to developing hyper perfusion syndrome after revascularization surgery [21]. A staged surgical approach is recommended to reduce this risk [22, 23]. The choice of anesthetics is also crucial; propofol or barbiturates have neuroprotective effects, helping to restore normal cerebral blood flow, making them safer options. In treatment, alleviating brain edema is also important, which can be achieved through the use of sedatives, mechanical ventilation, fever management, mannitol, hypertonic saline, and barbiturates [17, 24, 25]. Additionally, preoperative use of free radical scavengers significantly reduces the incidence of CHS and intracranial hemorrhage [26, 27, 28, 29].

For early prediction of postoperative CHS and intracranial hemorrhage risks, studies have shown that changes in middle cerebral artery flow velocity are strongly correlated with cerebral blood flow. Intraoperative and postoperative Transcranial Doppler (TCD) measurements have been proven to significantly enhance the accuracy of predicting CHS development after CEA [30]. Other imaging modalities, such as CT perfusion imaging, Laser Speckle Flowgraphy (LSFG), magnetic resonance perfusion imaging [31, 32], arterial spin labeling perfusion imaging, and single‐photon emission computed tomography (SPECT), can also serve as early predictive tools for hyper perfusion syndrome [33].

This case highlights the importance of identifying patients at high risk for CHS both before and after CAS. Early recognition and anticipation of CHS and intracranial hemorrhage, together with timely preventive measures, are particularly important in cerebrovascular patients with risk factors such as advanced age, hypertension, diabetes, and antiplatelet therapy. In addition to preoperative screening for high‐risk features, proactive postoperative blood pressure management and intensive monitoring are essential to prevent and limit severe hyper perfusion‐related complications. In high‐risk patients, strategies such as staged angioplasty, delayed intervention after ischemic stroke, and careful selection of anesthetic agents may further reduce the risk of CHS.

## Author Contributions

**Linling Ji:** conceptualization, data curation, writing – original draft. **Jiahao Wang:** data curation, writing – review and editing. **Yanyan Ji:** data curation, writing – review and editing. **Lin Zhu:** investigation, writing – review and editing. **Yingge Wang:** supervision, writing – review and editing.

## Funding

This work was supported by Yangzhou Science and Technology Bureau, [Grant No. YZ2023067]. The Postgraduate Research and Practice Innovation Program of Jiangsu Province, [Grant No. SJCX24_2352].

## Consent

All authors have read and approved the final manuscript and consent to its publication. Written informed consent was obtained from the patient's relative for the publication of this case report and any accompanying images. A copy of the written consent is available for review by the Editor of this journal upon request.

## Conflicts of Interest

The authors declare no conflicts of interest.

## Data Availability

Written informed consent was obtained from the patient for the publication of this case report and any accompanying images. The supporting data are available and can be provided to the journal for review upon request.
